# Supplementary material for: IL-1β Implications in Type 1 Diabetes Mellitus Progression: Systematic Review and Meta-Analysis
Source: J Clin Med. 2022 Feb 27;11(5):1303. doi: 10.3390/jcm11051303 (PMC8910979; doi:10.3390/jcm11051303)
Supplement: Supplementary file 1 [file jcm-11-01303-s001.zip › jcm-1592544-supplementary.pdf]

Table of contents

1. Search strategy 1
   1.1. Table S1. 1
2. List of full-text articles excluded with reasons 2
3. List of full-text articles included. 7
4. Characteristics of analyzed studies. 10
   4.1. Table S2. Variables of study. 10
5. Subgroup meta-analyses 11
   5.1. Figure S1. Geographical area. 11
   5.2. Figure S2. Age 12
   5.3. Figure S3. HbAc1 levels in patients <18 years 13
   5.4. Figure S4. Age matching 14
   5.5. Figure S5. Sex matching 15
   5.6. Figure S6. Source of sample 16
   5.7. Figure S7. Type of analysis 17
   5.8. Figure S8. Study design 18
6. Meta-regression analyses 19
   6.1. Figure S9. Effect of the covariate Sex 19
   6.2. Figure S10. Effect of the covariate Risk of Bias 20
7. Table S3. Sensitivity analysis. 21

1. **Search strategy**

1.1. Table S1.

Search strategy for each database, number of results, and execution date.

Database	Query/Search Strategy	Items founds/Results	Search time limits
PubMed	("diabetes mellitus, type 1"[MeSH Terms] OR "type 1 diabetes"[All Fields] OR "T1DM"[All Fields]) AND ("interleukin-1beta"[MeSH Terms] OR "il-1b"[All Fields] OR "il-1beta"[All Fields] OR "interleukin-1b"[All Fields] OR "interleukin-1beta"[All Fields])	626	October 25 - 2020
Embase	('type 1 diabetes' OR 'T1DM') AND ('interleukin 1beta'/exp OR 'il-1beta' OR 'il-1b' OR 'interleukin-1b')	817	October 25 - 2020
Web of Science	TS=("diabetes") AND TS=("type 1") AND TS=(interleukin 1 beta)	826	October 25 - 2020
Scopus	("diabetes mellitus,type 1" OR "type 1 diabetes" OR "T1DM") AND ("interleukin-1beta" OR "il-1b" OR "il-1beta" OR "interleukin-1b")	874	October 25 - 2020
Total	3,143		

2. List of full-text articles excluded with reasons

-Non control group

Al-Mubarak, S., Ciancio, S., Aljada, A., Mohanty, P., Mohanty, P., Ross, C., & Dandona, P. (2002). Comparative evaluation of adjunctive oral irrigation in diabetics. *Journal of Clinical Periodontology*, *29*(4), 295–300. https://doi.org/10.1034/j.1600-051X.2002.290404.x

Cherney, D. Z. I., Scholey, J. W., Daneman, D., Dunger, D. B., Dalton, R. N., Moineddin, R., Mahmud, F. H., Dekker, R., Elia, Y., Sochett, E., & Reich, H. N. (2012). Urinary markers of renal inflammation in adolescents with Type1 diabetes mellitus and normoalbuminuria. *Diabetic Medicine*, *29*(10), 1297–1302. https://doi.org/10.1111/j.1464-5491.2012.03651.x

Cherney, D. Z. I., Scholey, J. W., Sochett, E., Bradley, T. J., & Reich, H. N. (2011). The acute effect of clamped hyperglycemia on the urinary excretion of inflammatory cytokines/chemokines in uncomplicated type 1 diabetes: A pilot study. *Diabetes Care*, *34*(1), 177–180. https://doi.org/10.2337/dc10-1219

De Melo, E. N., Deda, L., Har, R., Reich, H. N., Scholey, J. W., Daneman, D., Moineddin, R., Motran, L., Elia, Y., Cherney, D. Z. I., Sochett, E. B., & Mahmud, F. H. (2016). The urinary inflammatory profile in gluten free diet - Adherent adolescents with type 1 diabetes and celiac disease. *Journal of Diabetes and Its Complications*, *30*(2), 295–299. https://doi.org/10.1016/j.jdiacomp.2015.11.020

Espersen, G. T., Mathiesen, O., Grunnet, N., Jensen, S., & DitzeL, J. (1993). Cytokine plasma levels and lymphocyte subsets in patients with newly diagnosed insulin-dependent (type 1) diabetes mellitus before and following initial insulin treatment. *Apmis*, *101*(7–12), 703–706. https://doi.org/10.1111/j.1699-0463.1993.tb00168.x

Gazizova, G., Gaysina, L., Valeeva, F., Abakumova, A., & Sharipova, J. (2017). Pregnancy Outcomes in Women with Type 1 Diabetes Depending on the Different Modes of Insulin Therapy. *BioNanoScience*, *7*(2), 390–395. https://doi.org/10.1007/s12668-016-0371-1

Gustavsson, C., Agardh, E., Bengtsson, B., & Agardh, C. D. (2008). TNF-α is an independent serum marker for proliferative retinopathy in type 1 diabetic patients. *Journal of Diabetes and Its Complications*, *22*(5), 309–316. https://doi.org/10.1016/j.jdiacomp.2007.03.001

Kadhim, K. A., Nafea, L. T., Fawzi, H. A., Hameed, E. A. Al, & Gasim, G. A. (2018). Assessment of vitamin d therapy effect on inflammatory markers in pediatric patients with type i diabetic. *Asian Journal of Pharmaceutical and Clinical Research*, *11*(10), 552–554. https://doi.org/10.22159/ajpcr.2018.v11i10.28936

Karavanaki, K., Kakleas, K., Georga, S., Bartzeliotou, A., Mavropoulos, G., Tsouvalas, M., Vogiatzi, A., Papassotiriou, I., & Karayianni, C. (2012). Plasma high sensitivity C-reactive protein and its relationship with cytokine levels in children with newly diagnosed type 1 diabetes and ketoacidosis. *Clinical Biochemistry*, *45*(16–17), 1383–1388. https://doi.org/10.1016/j.clinbiochem.2012.05.003

Karavanaki, K., Karanika, E., Georga, S., Bartzeliotou, A., Tsouvalas, M., Konstantopoulos, I., Fotinou, A., Papassotiriou, I., & Karayianni, C. (2011). Cytokine response to diabetic ketoacidosis (DKA) in children with type 1 diabetes (T1DM). *Endocrine Journal*, *58*(12), 1045–1053. https://doi.org/10.1507/endocrj.EJ11-0024

Kuehl, M. N., Rodriguez, H., Burkhardt, B. R., & Alman, A. C. (2015). Tumor necrosis factor-α, matrix-metalloproteinases 8 and 9 levels in the saliva are associated with increased hemoglobin A1c in type 1 diabetes subjects. *PLoS ONE*, *10*(4). https://doi.org/10.1371/journal.pone.0125320

Popescu, D., Gheorghe, D., Boldeanu, V., & Rica, A. M. (2017). Glycemic Control and Interleukin-1? in Periodontal Patients with Type 1 and 2 Diabetes in a Group of Subjects in South-West Romania. *Revista de Chimie*, *68*(12), 3002–3005. https://doi.org/10.37358/rc.17.12.6026

Rosa, J. S., Flores, R. L., Oliver, S. R., Pontello, A. M., Zaldivar, F. P., & Galassetti, P. R. (2008). Sustained IL-1α, IL-4, and IL-6 elevations following correction of hyperglycemia in children with type 1 diabetes mellitus. *Pediatric Diabetes*, *9*(1), 9–16. https://doi.org/10.1111/j.1399-5448.2007.00243.x

Schloot, N. C., Pham, M. N., Hawa, M. I., Pozzilli, P., Scherbaum, W., Schott, M., Kolb, H., Hunter, S., Schernthaner, G., Thivolet, C., Seissler, J., Leslie, R. D., Leslie, D., Beyan, H., Paschou, S. A., Williams, R., Brophy, S., Davies, H., BeckNielsen, H., … Mauricio, D. (2016). Inverse relationship between organ-specific autoantibodies and systemic immune mediators in type 1 diabetes and type 2 diabetes: Action LADA 11. *Diabetes Care*, *39*(11), 1932–1939. https://doi.org/10.2337/dc16-0293

- Lack of essential data

Fleiner, H. F., Radtke, M., Ryan, L., Moen, T., & Grill, V. (2014). Circulating immune mediators are closely linked in adult-onset type 1 diabetes as well as in non-diabetic subjects. *Autoimmunity*, *47*(8), 530–537. https://doi.org/10.3109/08916934.2014.938321

Kaštelan, S., Tomić, M., Pavan, J., & Orešković, S. (2010). Maternal immune system adaptation to pregnancy - a potential influence on the course of diabetic retinopathy. *Reproductive Biology and Endocrinology*, *8*. https://doi.org/10.1186/1477-7827-8-124

Lindehammer, S. R., Fex, M., Maziarz, M., Hanson, I., Maršál, K., & Lernmark, Å. (2011). Early-Pregnancy Cytokines in Mothers to Children Developing Multiple, Persistent Islet Autoantibodies, Type 1 Diabetes, or Both Before 7Years of Age. *American Journal of Reproductive Immunology*, *66*(6), 495–503. https://doi.org/10.1111/j.1600-0897.2011.01057.x

Maier, R., Weger, M., Haller-Schober, E. M., Huppertz, B., Maier, L. M., El-Shabrawi, Y., Wedrich, A., Theisl, A., Graninger, W., Demel, U., & Haas, A. (2010). Apoptotic death ligands and interleukins in the vitreous of diabetic patients *Spektrum Der Augenheilkunde*, *24*(6), 305–310. https://doi.org/10.1007/s00717-010-0446-2

Netea, M. G., Hancu, N., Blok, W. L., Grigorescu-Sido, P., Popa, L., Popa, V., & Van Der Meer, J. W. M. (1997). Interleukin 1β, tumour necrosis factor-α and interleukin 1 receptor antagonist in newly diagnosed insulin-dependent diabetes mellitus: Comparison to long-standing diabetes and healthy individuals. *Cytokine*, *9*(4), 284–287. https://doi.org/10.1006/cyto.1996.0165

Skogstrand, K., Thorsen, P., Nørgaard-Pedersen, B., Schendel, D. E., Sørensen, L. C., & Hougaard, D. M. (2005). Simultaneous measurement of 25 inflammatory markers and neurotrophins in neonatal dried blood spots by immunoassay with xMAP technology. *Clinical Chemistry*, *51*(10), 1854–1866. https://doi.org/10.1373/clinchem.2005.052241

Vistnes, M., Tapia, G., Mårild, K., Midttun, Ø., Ueland, P. M., Viken, M. K., Magnus, P., Berg, J. P., Gillespie, K. M., Skrivarhaug, T., Njølstad, P. R., Joner, G., Størdal, K., & Stene, L. C. (2018). Plasma immunological markers in pregnancy and cord blood: A possible link between macrophage chemo-attractants and risk of childhood type 1 diabetes. *American Journal of Reproductive Immunology*, *79*(3). https://doi.org/10.1111/aji.12802

- No IL1B

Barat, P., Brossaud, J., Lacoste, A., Vautier, V., Nacka, F., Moisan, M. P., & Corcuff, J. B. (2013). Nocturnal activity of 11β-hydroxy steroid dehydrogenase type 1 is increased in type 1 diabetic children. *Diabetes and Metabolism*, *39*(2), 163–168. https://doi.org/10.1016/j.diabet.2012.10.001

Özer, G., Teker, Z., Çetiner, S., Yilmaz, M., Topaloglu, A. K., Önenli-Mungan, N., & Yüksel, B. (2003). Serum IL-1, IL-2, TNFα and INFγ levels of patients with type 1 diabetes mellitus and their siblings. *Journal of Pediatric Endocrinology and Metabolism*, *16*(2), 203–210. https://doi.org/10.1515/JPEM.2003.16.2.203

Strom, A., Menart, B., Simon, M. C., Pham, M. N., Kolb, H., Roden, M., Pozzilli, P., Leslie, R. D. G., & Schloot, N. C. (2012). Cellular interferon-γ and interleukin-13 immune reactivity in type 1, type 2 and latent autoimmune diabetes: Action LADA 6. *Cytokine*, *58*(2), 148–151. https://doi.org/10.1016/j.cyto.2012.01.002

Waugh, K., Snell-Bergeon, J., Michels, A., Dong, F., Steck, A. K., Frohnert, B. I., Norris, J. M., & Rewers, M. (2017). Increased inflammation is associated with islet autoimmunity and type 1 diabetes in the Diabetes Autoimmunity Study in the Young (DAISY). *PLoS ONE*, *12*(4). https://doi.org/10.1371/journal.pone.0174840

- In vitro

Cheung, A. T. W., Tomic, M. M., Chen, P. C. Y., Miguelino, E., Li, C. S., & Devaraj, S. (2009). Correlation of microvascular abnormalities and endothelial dysfunction in Type-1 diabetes mellitus (T1DM): A real-time intravital microscopy study. *Clinical Hemorheology and Microcirculation*, *42*(4), 285–295. https://doi.org/10.3233/CH-2009-1199

Devaraj, S., Glaser, N., Griffen, S., Wang-Polagruto, J., Miguelino, E., & Jialal, I. (2006). Increased monocytic activity and biomarkers of inflammation in patients with type 1 diabetes. *Diabetes*, *55*(3), 774–779. https://doi.org/10.2337/diabetes.55.03.06.db05-1417

- No type 1 diabetes

Shahzad, K., Bock, F., Wang, H., Kopf, S., Wacker, C., Kohli, S., Wolter, J., Ranjan, S., Reymann, K., Stoyanov, S., Groene, H., Madhusudhan, T., Nawroth, P., & Isermann, B. (2014). Activation of the Nlrp3 inflammasome via mitochondrial ROS in glomerular cells aggravates experimental diabetic nephropathy. *Diabetologie Und Stoffwechsel*, *9*(S 01). https://doi.org/10.1055/s-0034-1375018

Svensson, J., Oderup, C., Åkesson, C., Uvebrant, K., Hallengren, B., Ericsson, U. B., Arvastsson, J., Danska, J. S., Lantz, M., & Cilio, C. M. (2011). Maternal autoimmune thyroid disease and the fetal immune system. *Experimental and Clinical Endocrinology and Diabetes*, *119*(7), 445–450. https://doi.org/10.1055/s-0031-1279741

-Off topic

Anquetil, F., Sabouri, S., Thivolet, C., Rodriguez-Calvo, T., Zapardiel-Gonzalo, J., Amirian, N., Schneider, D., Castillo, E., Lajevardi, Y., & von Herrath, M. G. (2017). Alpha cells, the main source of IL-1β in human pancreas. *Journal of Autoimmunity*, *81*, 68–73. https://doi.org/10.1016/j.jaut.2017.03.006

Chen, Y. G., Cabrera, S. M., Jia, S., Kaldunski, M. L., Kramer, J., Cheong, S., Geoffrey, R., Roethle, M. F., Woodliff, J. E., Greenbaum, C. J., Wang, X., & Hessner, M. J. (2014). Molecular signatures differentiate immune states in type 1 diabetic families. *Diabetes*, *63*(11), 3960–3973. https://doi.org/10.2337/db14-0214

- Overlapping population

Kyvsgaard, J. N., Ellervik, C., Lindkvist, E. B., Pipper, C. B., Pociot, F., Svensson, J., & Thorsen, S. U. (2019). Perinatal whole blood zinc status and cytokines, adipokines, and other immune response proteins. *Nutrients*, *11*(9). https://doi.org/10.3390/nu11091980

Sildorf, S. M., Eising, S., Hougaard, D. M., Mortensen, H. B., Skogstrand, K., Pociot, F., Johannesen, J., & Svensson, J. (2014). Differences in MBL levels between juvenile patients newly diagnosed with type 1 diabetes and their healthy siblings. *Molecular Immunology*, *62*(1), 71–76. https://doi.org/10.1016/j.molimm.2014.06.001

3. List of full-text articles included.

Abdel-Latif, M., Abdel-Moneim, A. A., El-Hefnawy, M. H., & Khalil, R. G. (2017). Comparative and correlative assessments of cytokine, complement and antibody patterns in paediatric type 1 diabetes. *Clinical and Experimental Immunology*, *190*(1), 110–121. https://doi.org/10.1111/cei.13001

Aguilera, E., Serra-Planas, E., Granada, M. L., Pellitero, S., Reverter, J. L., Alonso, N., … Puig-Domingo, M. (2015). Relationship of YKL-40 and adiponectin and subclinical atherosclerosis in asymptomatic patients with type 1 diabetes mellitus from a European Mediterranean population. *Cardiovascular Diabetology*, *14*(1), 1–7. https://doi.org/10.1186/s12933-015-0287-z

Akopova, O. V., Kolchinskaya, L. I., Nosar, V. I., Bouryi, V. A., Mankovska, I. N., & Sagach, V. F. (2012). Cytochrome C as an amplifier of ROS release in mitochondria. *Fiziolohichnyĭ Zhurnal (Kiev, Ukraine : 1994)*, *58*(1), 3–12. https://doi.org/10.15407/fz58.01.003

Allam, G., Alsulaimani, A. A., Alghamdi, H., Alswat, H., Edrees, B. M., Ahmad, I., & Nasr, A. (2014). Changes in the levels of cytokines in both diabetic/non-diabetic type i children living in a moderate altitude area in Saudi Arabia. *High Altitude Medicine and Biology*, *15*(3), 380–387. https://doi.org/10.1089/ham.2014.1001

Alnek, K., Kisand, K., Heilman, K., Peet, A., Varik, K., & Uibo, R. (2015). Increased blood levels of growth factors, proinflammatory cytokines, and Th17 cytokines in patients with newly diagnosed type 1 diabetes. *PLoS ONE*, *10*(12), 1–16. https://doi.org/10.1371/journal.pone.0142976

Andrade Lima Gabbay, M., Sato, M. N., Duarte, A. J. S., & Dib, S. A. (2012). Serum titres of anti-glutamic acid decarboxylase-65 and anti-IA-2 autoantibodies are associated with different immunoregulatory milieu in newly diagnosed type 1 diabetes patients. *Clinical and Experimental Immunology*, *168*(1), 60–67. https://doi.org/10.1111/j.1365-2249.2011.04538.x

Aravindhan, V., Mohan, V., Arunkumar, N., Sandhya, S., & Babu, S. (2015). Chronic endotoxemia in subjects with type-1 diabetes is seen much before the onset of microvascular complications. *PLoS ONE*, *10*(9), 1–11. https://doi.org/10.1371/journal.pone.0137618

Aribi, M., Moulessehoul, S., Kendouci-Tani, M., Benabadji, A. B., Hichami, A., & Khan, N. A. (2007). Relationship between interleukin-1β and lipids in type 1 diabetic patients. *Medical Science Monitor*, *13*(8), 372–378.

Dogan, Y., Akarsu, S., Ustundag, B., Yilmaz, E., & Gurgoze, M. K. (2006). Serum IL-1β, IL-2, and IL-6 in insulin-dependent diabetic children. *Mediators of Inflammation*, *2006*, 1–6. https://doi.org/10.1155/MI/2006/59206

Duarte, P. M., Neto, J. B. C., Casati, M. Z., Sallum, E. A., & Nociti, F. H. (2007). Diabetes modulates gene expression in the gingival tissues of patients with

chronic periodontitis. *Oral Diseases*, *13*(6), 594–599. https://doi.org/10.1111/j.1601-0825.2006.01348.x

Farhan, J., Al-Shobaili, H. A., Zafar, U., Al Salloom, A., Meki, A. R., & Rasheed, Z. (2014). Interleukin-6: A possible inflammatory link between vitiligo and type 1 diabetes. *British Journal of Biomedical Science*, *71*(4), 151–157. https://doi.org/10.1080/09674845.2014.11669980

Fatima, N., Faisal, S. M., Zubair, S., Ajmal, M., Siddiqui, S. S., Moin, S., & Owais, M. (2016). Role of pro-inflammatory cytokines and biochemical markers in the pathogenesis of type 1 diabetes: Correlation with age and glycemic condition in diabetic human subjects. *PLoS ONE*, *11*(8), 1–17. https://doi.org/10.1371/journal.pone.0161548

Ferreira, M., João, D., & Alessandra, G. (2017). Microbiological , lipid and immunological SUR ¿ OHV LQ FKLOGUHQ ZLWK JLQJLYLWLV DQG type 1 diabetes mellitus. *Journal of Applied Oral Science*, *25*(2), 217–226.

Holm, B. C., Svensson, J., Åkesson, C., Arvastsson, J., Ljungberg, J., Lynch, K., … Cilio, C. M. (2006). Evidence for immunological priming and increased frequency of CD4 + CD25+ cord blood T cells in children born to mothers with type 1 diabetes. *Clinical and Experimental Immunology*, *146*(3), 493–502. https://doi.org/10.1111/j.1365-2249.2006.03243.x

Koskela, U. E., Kuusisto, S. M., Nissinen, A. E., Savolainen, M. J., & Liinamaa, M. J. (2013). High vitreous concentration of IL-6 and IL-8, but not of adhesion molecules in relation to plasma concentrations in proliferative diabetic retinopathy. *Ophthalmic Research*, *49*(2), 108–114. https://doi.org/10.1159/000342977

Leiva-Gea, I., Sánchez-Alcoholado, L., Martín-Tejedor, B., Castellano-Castillo, D., Moreno-Indias, I., Urda-Cardona, A., … Queipo-Ortuño, M. I. (2018). Gut microbiota differs in composition and functionality between children with type 1 diabetes and MODY2 and healthy control subjects: A case-control study. *Diabetes Care*, *41*(11), 2385–2395. https://doi.org/10.2337/dc18-0253

Lo, H. C., Lin, S. C., & Wang, Y. M. (2004). The relationship among serum cytokines, chemokine, nitric oxide, and leptin in children with type 1 diabetes mellitus. *Clinical Biochemistry*, *37*(8), 666–672. https://doi.org/10.1016/j.clinbiochem.2004.02.002

Meyers, A. J., Shah, R. R., Gottlieb, P. A., & Zipris, D. (2010). Altered toll-like receptor signaling pathways in human type 1 diabetes. *Journal of Molecular Medicine*, *88*(12), 1221–1231. https://doi.org/10.1007/s00109-010-0666-6

Mohamed, W. A., Abdelhamid, N., Selim, A., & Salama, E. (2016). Effect of raw camel milk on proinflammatoryadipocytokines levels in children with type 1 diabetes mellitus. *Research Journal of Pharmaceutical, Biological and Chemical Sciences*, *7*(4), 1096–1101.

Pérez B, F., Oyarzún A, A., Carrasco P, E., Angel B, B., Albala B, C., & Santos M, J. L. (2004). Niveles plasmáticos de citoquinas IL-1β, IL2 e IL-4 en niños

diabéticos tipo 1 de diagnóstico reciente y su asociación con anticuerpos β pancreáticos. *Revista Medica de Chile*, *132*(4), 413–420. https://doi.org/10.4067/s0034-98872004000400002

Salvi, G. E., Franco, L. M., Braun, T. M., Lee, A., Rutger Persson, G., Lang, N. P., & Giannobile, W. V. (2010). Pro-inflammatory biomarkers during experimental gingivitis in patients with type 1 diabetes mellitus: A proof-of-concept study. *Journal of Clinical Periodontology*, *37*(1), 9–16. https://doi.org/10.1111/j.1600-051X.2009.01500.x

Svensson, J., Eising, S., Hougaard, D. M., Mortensen, H. B., Skogstrand, K., Simonsen, L. B., … Johannesen, J. (2012). Few differences in cytokines between patients newly diagnosed with type 1 diabetes and their healthy siblings. *Human Immunology*, *73*(11), 1116–1126. https://doi.org/10.1016/j.humimm.2012.07.337

Talaat, I. M., Nasr, A., Alsulaimani, A. A., Alghamdi, H., Alswat, K. A., Almalki, D. M., … Allam, G. (2016). Association between type 1, type 2 cytokines, diabetic autoantibodies and 25-hydroxyvitamin D in children with type 1 diabetes. *Journal of Endocrinological Investigation*, *39*(12), 1425–1434. https://doi.org/10.1007/s40618-016-0514-9

Thorsen, S. U., Pipper, C. B., Ellervik, C., Pociot, F., Kyvsgaard, J. N., & Svensson, J. (2019). Association between neonatal whole blood iron content and cytokines, adipokines, and other immune response proteins. *Nutrients*, *11*(3), 1–12. https://doi.org/10.3390/nu11030543

Ururahy, M. A. G., Loureiro, M. B., Freire-Neto, F. P., de Souza, K. S. C., Zuhl, I., Brandão-Neto, J., … de Rezende, A. A. (2012). Increased TLR2 expression in patients with type 1 diabetes: Evidenced risk of microalbuminuria. *Pediatric Diabetes*, *13*(2), 147–154. https://doi.org/10.1111/j.1399-5448.2011.00794.x

Ziaja, J., Kowalik, A. P., Kolonko, A., Kamińska, D., Owczarek, A. J., Kujawa-Szewieczek, A., … Cierpka, L. (2018). Type 1 diabetic patients have better endothelial function after simultaneous pancreas–kidney transplantation than after kidney transplantation with continued insulin therapy. *Diabetes and Vascular Disease Research*, *15*(2), 122–130. https://doi.org/10.1177/1479164117744423

4. Characteristics of analyzed studies.

4.1. Table S2. Variables of study.

Study	Year	Country	Population (n) (Male, Female)		Age (Years)		Sample	IL1-β (pg/ml or mRNA/GADPH)		Technique	Evolution (years)	HbA1c%	Study design
			T1DM	Control	T1DM Mean (SD)	Control Mean (SD)		T1DM Mean (SD)	Control Mean (SD)		Mean (SD)	Mean (SD)	
Pérez-Bravo *et al.*	2004	Chile	120 (66, 54)	118 (62, 56)	8.5 (3.5)	9.1 (5.5)	Serum	9.4 (7.3)	4.96 (4)	ELISA	NA	NA	CC
Lo *et al.*	2004	Taiwan	58 (22, 36)	33 (16, 17)	10.98 (4.61)	10.06 (4.9)	Serum	46.43 (79.71)	52.98 (82.13)	ELISA	NA	8.7 (1.59)	CC
Holm *et al.*	2006	Sweden	13 (NA, NA)	82 (NA, NA)	0.72 (1)	0.77 (1.6)	CBP	178.60 (236.73)	52.77 (106.68)	FC	NA	NA	CC
Dogan *et al.*	2006	Turkey	27 (15, 12)	25 (14, 11)	10.05 (1.85)	10.5 (1)	Serum	12.86 (2.27)	3.8 (0.9)	ELISA	NA	8.95 (1.4)	CC
Arabi *et al.*	2007	Algeria	69 (29, 40)	74 (36, 38)	15.3 (0.32)	15.71 (0.37)	Plasma	3 (3.32)	0.5 (5.16)	ELISA	13.41 (0.25)	9.5 (0.83)	CC
Duarte *et al.*	2007	Brazil	20 (7, 13)	20 (8, 12)	50.19 (11.41)	49.5 (8.11)	GB	1.16 (0.7)	2.61 (0.83)	RT-PCR	12.06 (7.52)	NA	CC
Salvi *et al.*	2010	Switzerland	9 (NA, NA)	9 (NA, NA)	25.6 (5.8)	24.8 (5.7)	GCF	3130.09 (653.53)	1444.66 (275.17)	ELISA	9.0 (5.3)	8.1 (0.7)	C
Meyers *et al.*	2010	USA	20 (NA, NA)	18 (NA, NA)	16.2 (6.6)	12.9 (3.4)	Serum	48.49 (30.86)	32.89 (56.99)	IP	0.19	8.8 (2.7)	CC
Gabbay *et al.*	2012	Brazil	35 (20, 15)	25 (16, 9)	13 (5)	13.6 (5.4)	Serum	1 (3)	0.01 (0.01)	FC	NA	8.4 (2.2)	CC
Svensson *et al.*	2012	Denmark	482 (255, 227)	479 (266, 213)	9.83	10.2	Serum	5115.69 (1418.09)	4940.51 (1257.73)	IP	NA	NA	CC
Ururahya *et al.*	2012	Brazil	76 (23, 53)	100 (43, 57)	12.2 (3.8)	11.9 (3.6)	PB	1.86 (0.9)	1.44 (0.72)	RT-PCR	5.3 (3.7)	10.26 (3.1)	CC
Fartushok *et al.*	2012	Ukraine	25 (10, 15)	10 (NA, NA)	35	35	Plasma	11.71 (0.69)	3 (0.62)	ELISA	NA	NA	CC
Koskela *et al.*	2013	Finland	38 (17, 21)	16 (5, 11)	59.4 (14.3)	66.6 (9.3)	Plasma	29.9 (63.5)	66.7 (108)	ELISA	24.7 (12.3)	8.5 (1.5)	CSS
							Vitreo	0.3 (0.9)	1.2 (2.4)				
Allam *et al.*	2014	Saudi Arabia	150 (75, 75)	150 (75, 75)	9.82	10.19	Serum	9.54 (3.43)	2.45 (1.13)	FC	NA	9.45 (2.9)	CSS
Farhan *et al.*	2014	Saudi Arabia	37 (18, 19)	42 (27, 15)	33.1 (16.2)	43 (17)	Serum	1.57 (0.504)	0.34 (0.23)	ELISA	NA	NA	
Aguilera *et al.*	2015	Spain	150 (87, 63)	50 (28, 22)	38.6 (8.1)	38.1 (7.2)	Serum	1 (1.1)	1.1 (0.8)	IP	20.4 (8.1)	8.1 (2.3)	CC
Aravindhan *et al.*	2015	India	97 (49, 48)	64 (32, 32)	29.02 (16.94)	30.4 (15.76)	Serum	15.21 (14.36)	9.88 (14.34)	ELISA	8.23 (7.14)	10.7 (3.8)	CC
Alnek *et al.*	2015	Estonia	36 (17, 19)	20 (8, 12)	9.53 (5.94)	13.87 (10.85)	Plasma	6.36 (3.32)	3.24 (3.19)	IP	<1	NA	CC
Mohamed *et al.*	2016	Egypt	30 (NA, NA)	30 (NA, NA)	8.8 (2.85)	9.83 (2.81)	Serum	7.763 (1.119)	3.403 (0.88)	ELISA	NA	7.067 (0.872)	CC
Fatima *et al.*	2016	India	29 (17, 12)	25 (NA, NA)	14.25 (4)	14.25 (4)	Serum	18.46 (3.5)	4.04 (0.2)	ELISA	NA	9.17 (1.33)	CC
Talaat *et al.*	2016	Saudi Arabia	250 (NA, NA)	250 (NA, NA)	8.5 (0.5)	8.5 (0.5)	Serum	12.09 (2.94)	3.39 (0.5)	ELISA	3.5 (4.69)	11.35 (3.1)	CSS
Duque *et al.*	2017	Brazil	24 (12, 12)	27 (13, 14)	9.45 (1.69)	9.62 (1.86)	Serum	1.57 (0.31)	1.98 (0.29)	ELISA	NA	6.94 (1.58)	CC
Abdel-Latif *et al.*	2017	Egypt	100 (50, 50)	100 (50, 50)	9.8 (2.9)	9.1 (2.7)	Serum	12.46 (0.37)	2.56 (0.366)	ELISA	NA	8.87 (1.07)	CC
Leiva-Gea *et al.*	2018	Spain	15 (7, 8)	13 (7, 6)	12.56 (3.59)	12.25 (2.92)	Serum	119.41 (27.12)	83.21 (28.25)	ELISA	5.68 (1.84)	6.26 (0.38)	CC
Ziaja *et al.*	2018	Poland	39 (17, 22)	52 (23, 29)	48.8 (9.1)	44.8 (12)	Plasma	2.67 (3.92)	1.3 (1.6)	ELISA	35.8 (8.6)	7.7 (1.1)	CSS
Thorsen *et al.*	2019	Denmark	199 (96, 103)	199 (104, 95)	Neonatal	Neonatal	Serum	176.17 (112.39)	172.34 (120.22)	ELISA	NA	NA	C

Abbreviation: T1DM, Type 1 Diabetes Mellitus; SD, Standard Deviation; IL1β, Interleukin-1 beta; mRNA, messenger ribonucleic acid; GADPH, glyceraldehyde-3-phosphate dehydrogenase; HbA1c, Glycated hemoglobin A1c; ELISA, Enzyme-Linked ImmunoSorbent Assay; NA, Non Available; CC, case control; CBP, Cord Blood Plasma; FC, Flow cytometry; GB, Gingival biopsies; RT-PCR, Reverse transcription polymerase chain reaction; GCF, Gingival crevicular fluid; IP, Innmunoassay panel; C, cohort; PB, Peripheral blood; CSS, cross-sectional study.

5. Subgroup meta-analyses

5.1. Figure S1. Geographical area.

Forest plot graphically representing the meta-analyses evaluating the changes in circulating IL-1β levels (immunoassays determination) between T1DM patients and controls by geographical area.

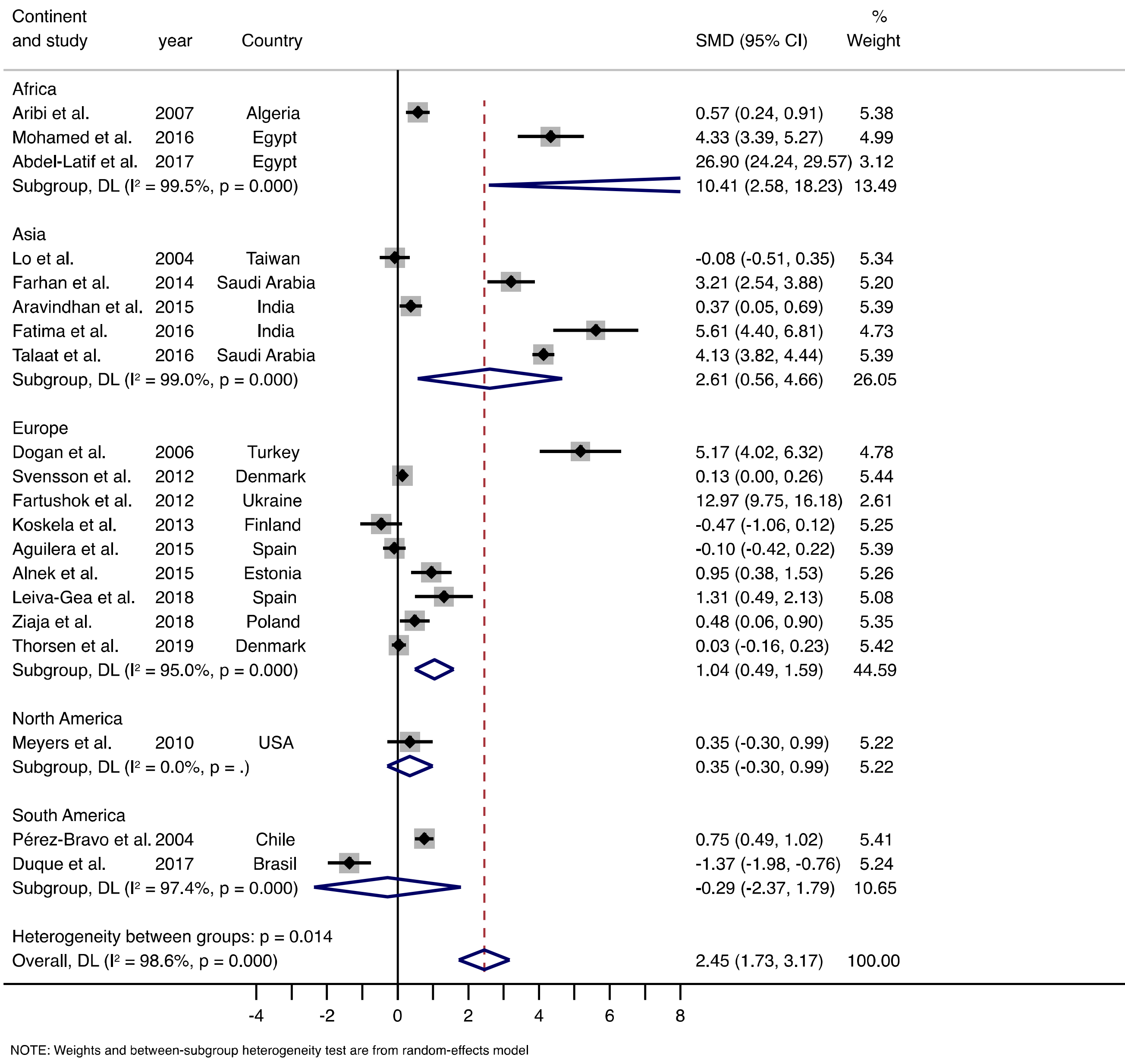

Random-effects model, inverse-variance weighting based on the DerSimonian and Laird method. Standardized mean difference (SMD) was chosen as effect size measure. An SMD>0 suggests that IL-1β levels are higher in T1DM. Diamonds indicate the overall pooled SMDs with their corresponding 95% confidence intervals (CI).

5.2. Figure S2. Age

Forest plot graphically representing the meta-analyses evaluating the changes in circulating IL-1β levels (immunoassays determination) between T1DM patients and controls by age (<18, children vs >18 years old, adults).

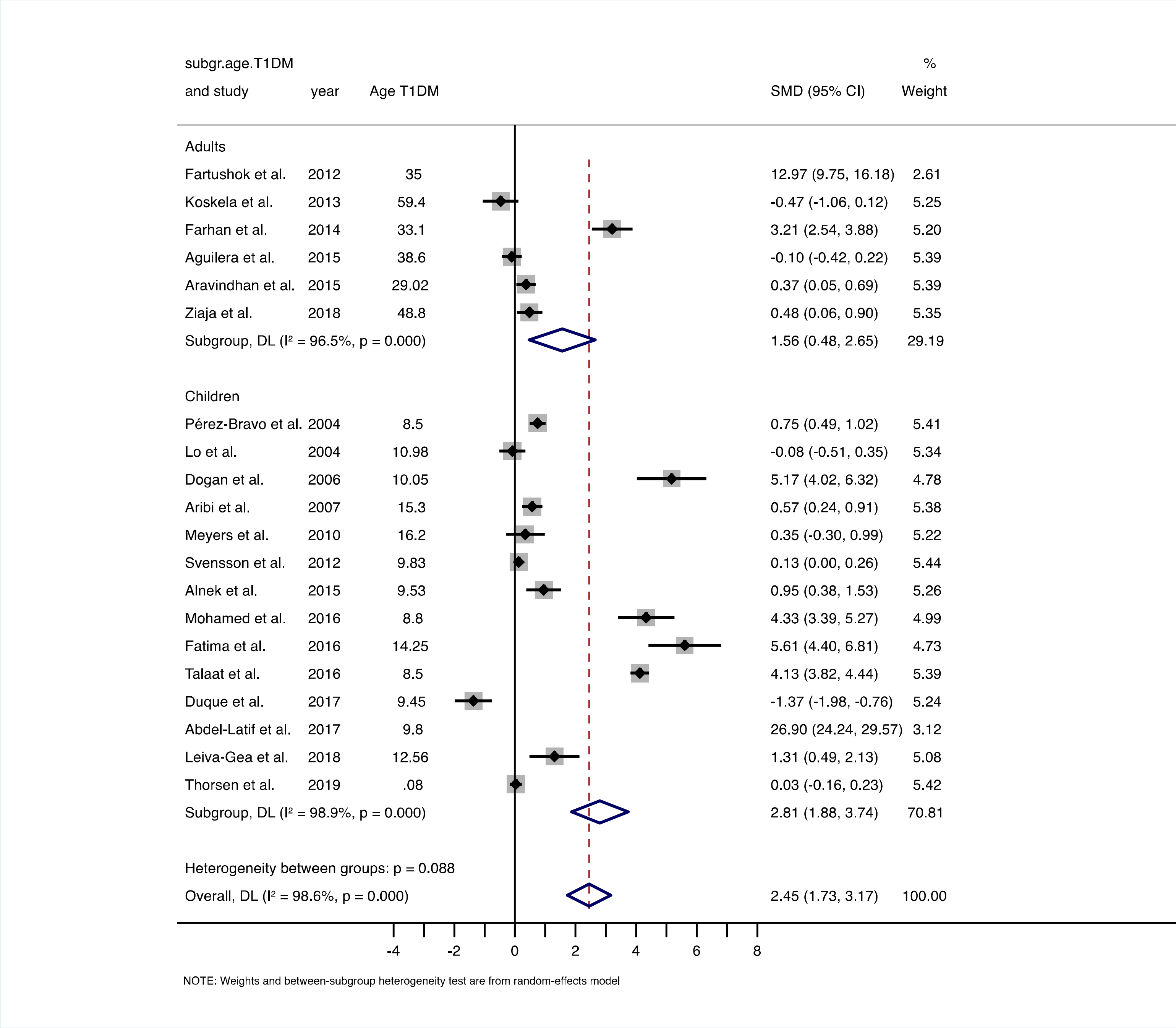

Random-effects model, inverse-variance weighting based on the DerSimonian and Laird method. Standardized mean difference (SMD) was chosen as effect size measure. An SMD>0 suggests that IL-1β levels are higher in T1DM. Diamonds indicate the overall pooled SMDs with their corresponding 95% confidence intervals (CI).

5.3. Figure S3. HbAc1 levels in patients <18 years

Forest plot graphically representing the meta-analyses evaluating the changes in circulating IL-1β levels (immunoassays determination) between T1DM patients and controls by HbAc1levels in patients <18 years (<7 vs >7).

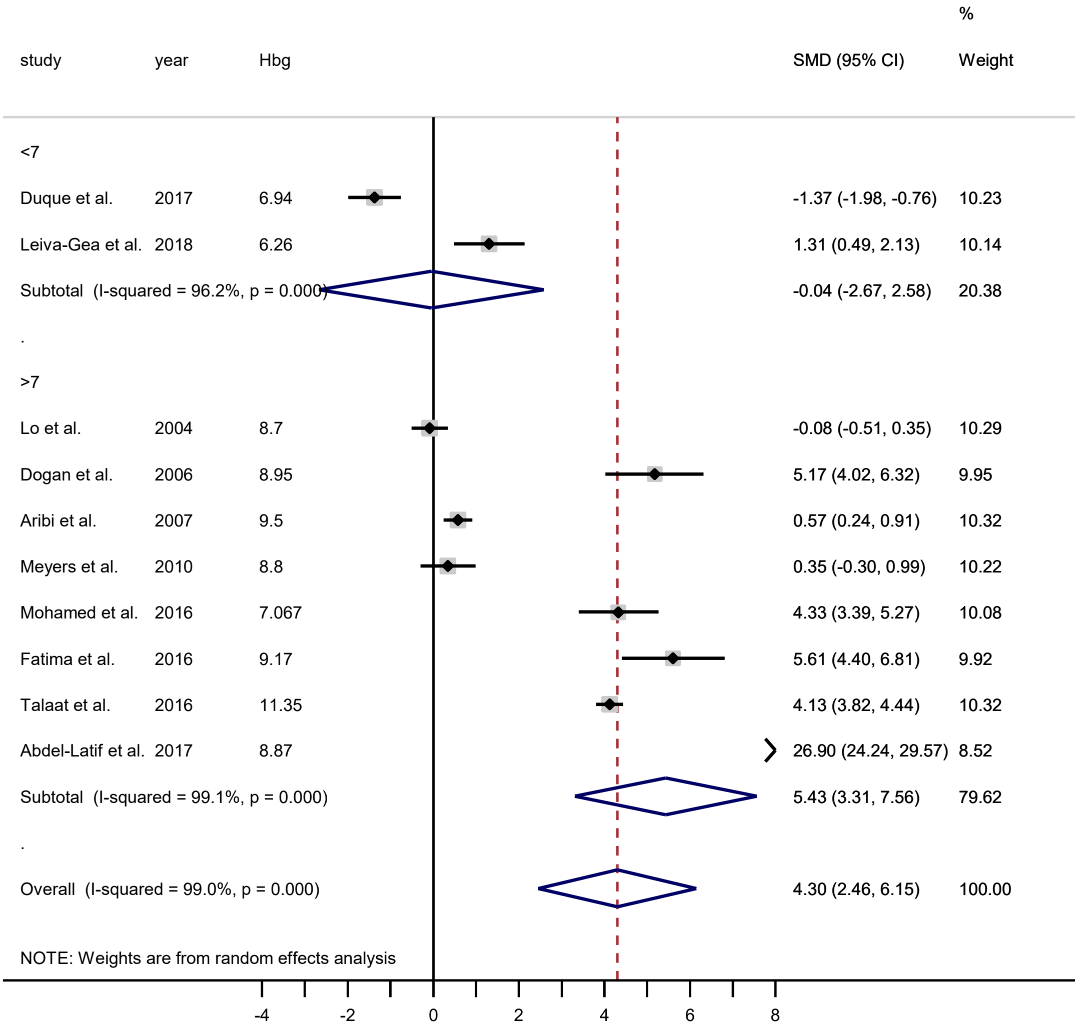

Random-effects model, inverse-variance weighting based on the DerSimonian and Laird method. Standardized mean difference (SMD) was chosen as effect size measure. An SMD>0 suggests that IL-1β levels are higher in T1DM. Diamonds indicate the overall pooled SMDs with their corresponding 95% confidence intervals (CI).

5.4. Figure S4. Age matching

Forest plot graphically representing the meta-analyses evaluating the changes in circulating IL-1β levels (immunoassays determination) between T1DM patients and controls by age matching (matched vs unmatched).

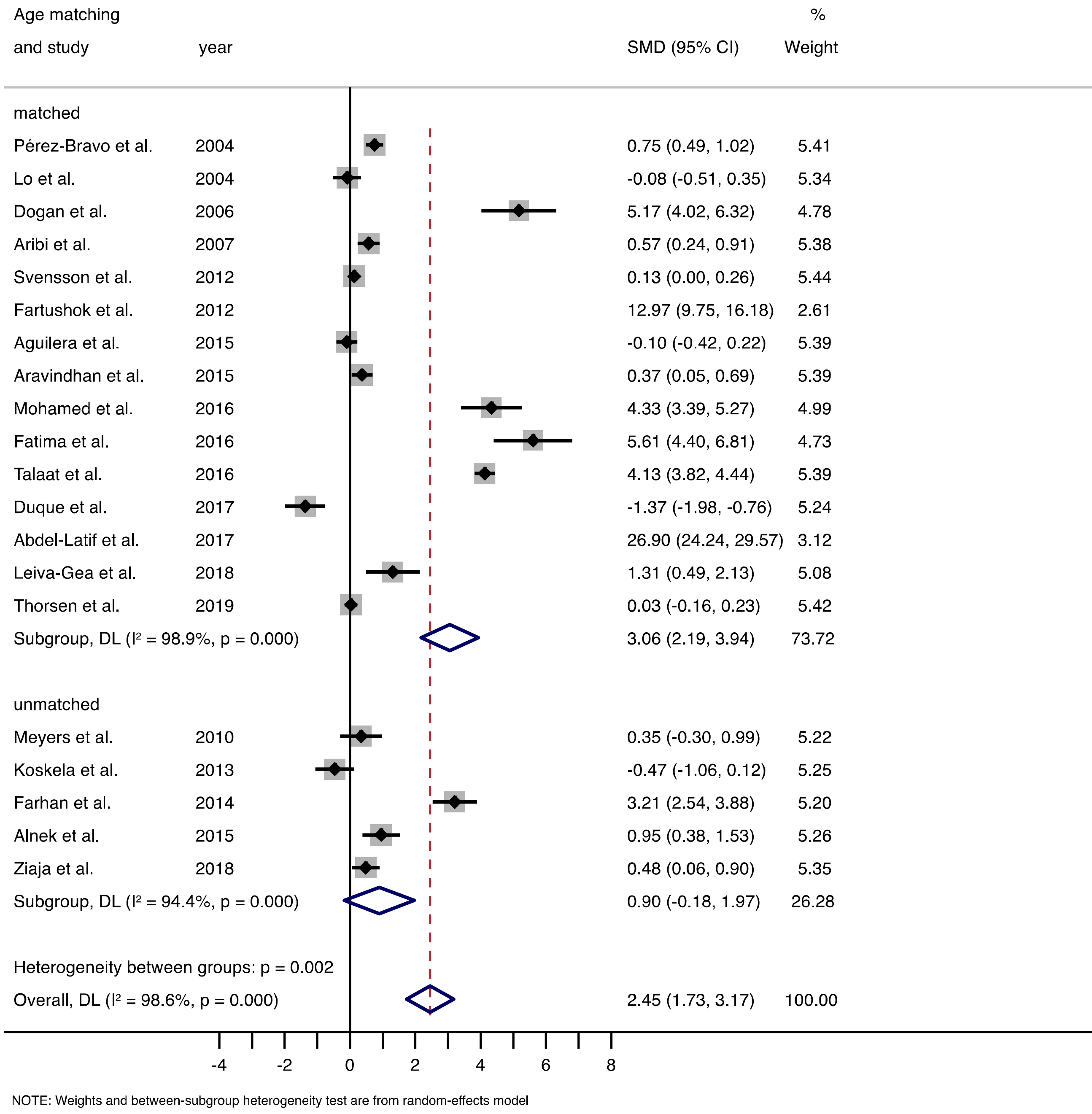

Random-effects model, inverse-variance weighting based on the DerSimonian and Laird method. Standardized mean difference (SMD) was chosen as effect size measure. An SMD>0 suggests that IL-1β levels are higher in T1DM. Diamonds indicate the overall pooled SMDs with their corresponding 95% confidence intervals (CI).

5.5. Figure S5. Sex matching

Forest plot graphically representing the meta-analyses evaluating the changes in circulating IL-1β levels (immunoassays determination) between T1DM patients and controls by sex matching (matched vs unmatched).

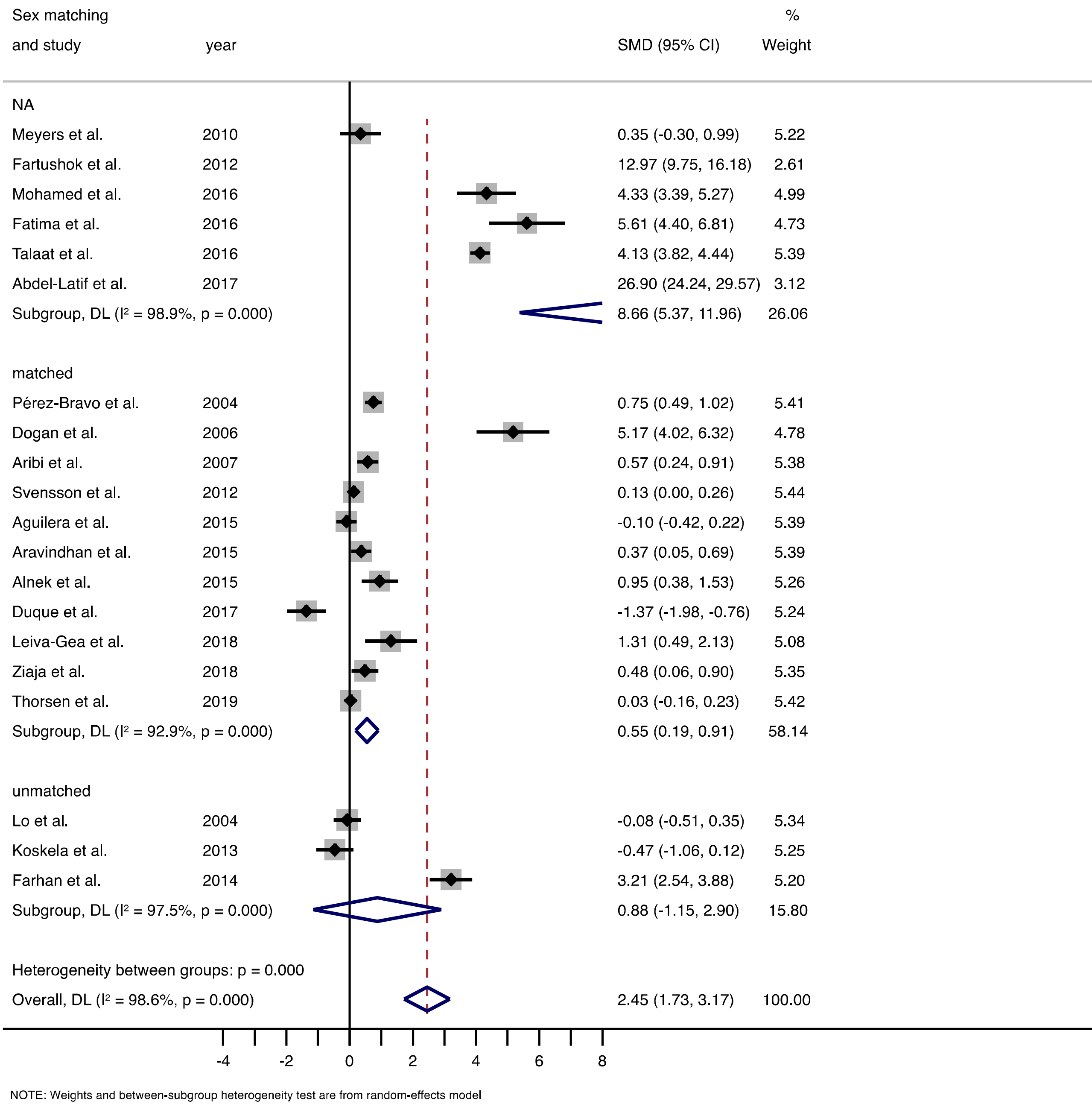

Random-effects model, inverse-variance weighting based on the DerSimonian and Laird method. Standardized mean difference (SMD) was chosen as effect size measure. An SMD>0 suggests that IL-1β levels are higher in T1DM. Diamonds indicate the overall pooled SMDs with their corresponding 95% confidence intervals (CI).

5.6. Figure S6. Source of sample

Forest plot graphically representing the meta-analyses evaluating the changes in circulating IL-1β levels (immunoassays determination) between T1DM patients and controls by source of sample (Serum, Plasma, Gingival crevicular fluid or Vitreous humour).

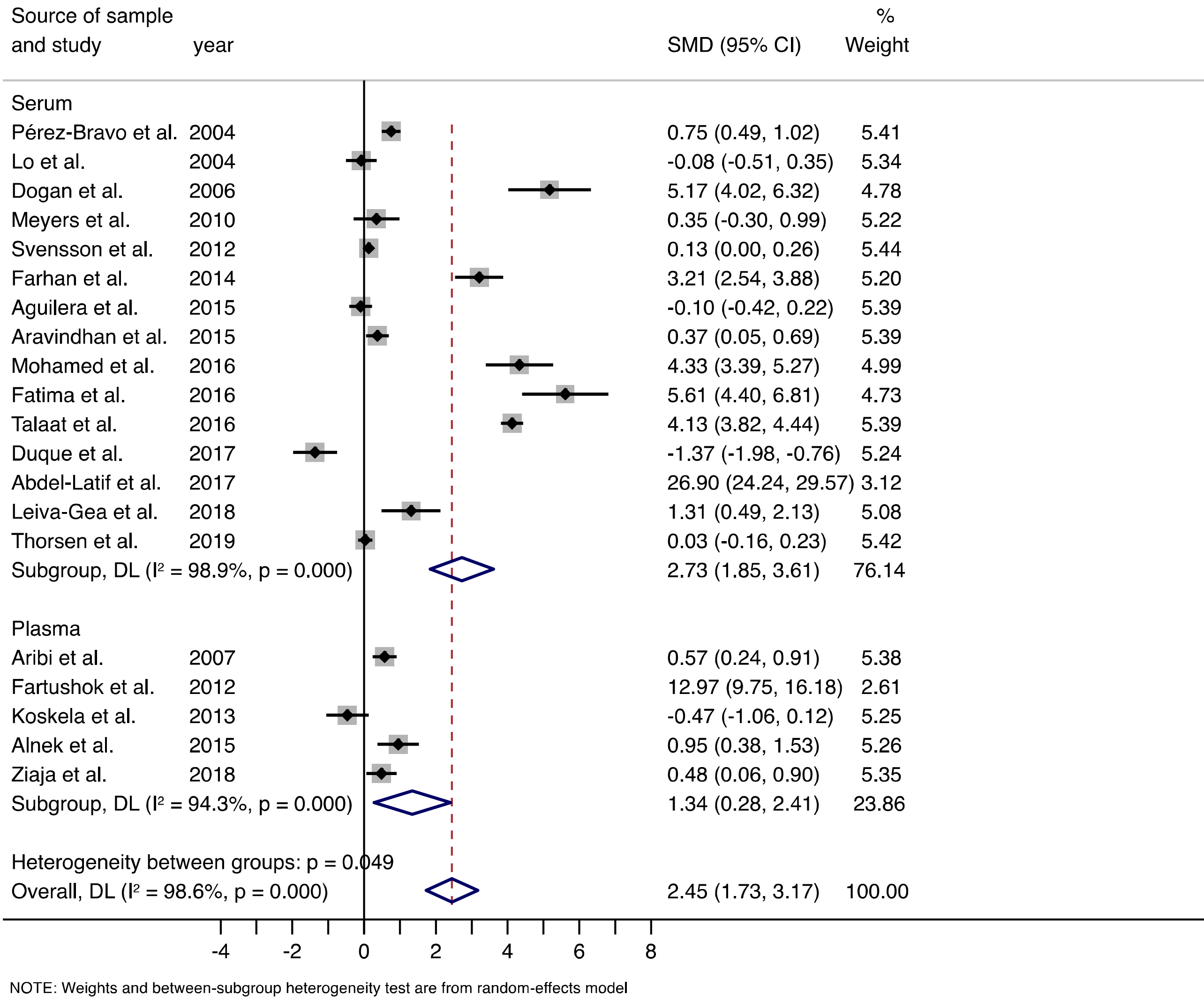

Random-effects model, inverse-variance weighting based on the DerSimonian and Laird method. Standardized mean difference (SMD) was chosen as effect size measure. An SMD>0 suggests that IL-1β levels are higher in T1DM. Diamonds indicate the overall pooled SMDs with their corresponding 95% confidence intervals (CI).

5.7. Figure S7. Type of analysis

Forest plot graphically representing the meta-analyses evaluating the changes in circulating IL-1β levels (immunoassays determination) between T1DM patients and controls by type of analysis (ELISA vs immunoassay panel).

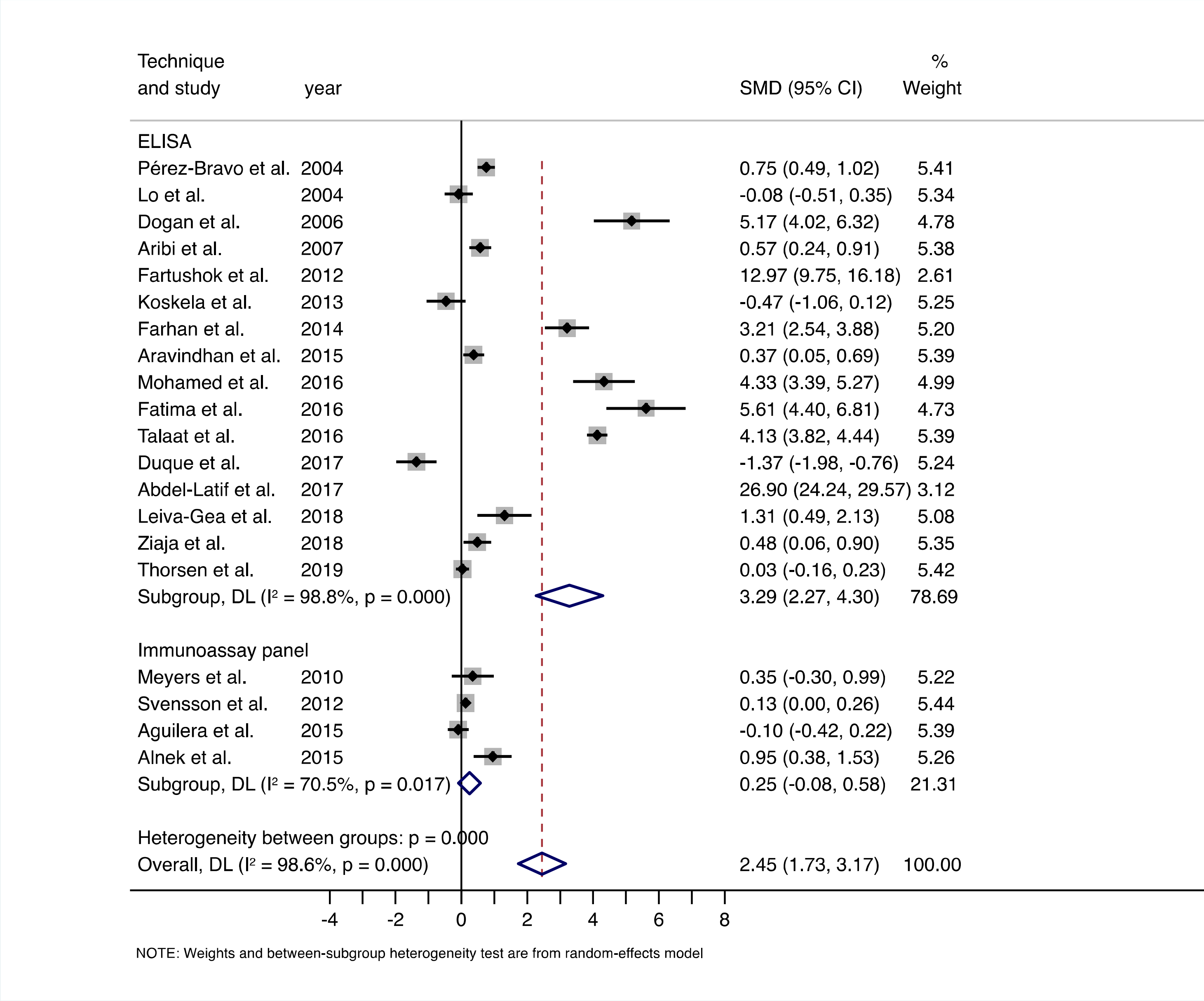

Random-effects model, inverse-variance weighting based on the DerSimonian and Laird method. Standardized mean difference (SMD) was chosen as effect size measure. An SMD>0 suggests that IL-1β levels are higher in T1DM. Diamonds indicate the overall pooled SMDs with their corresponding 95% confidence intervals (CI).

5.8. Figure S8. Study design

Forest plot graphically representing the meta-analyses evaluating the changes in circulating IL-1β levels (immunoassays determination) between T1DM patients and controls by study design (Case-control, or Cohort, or Cross-sectional).

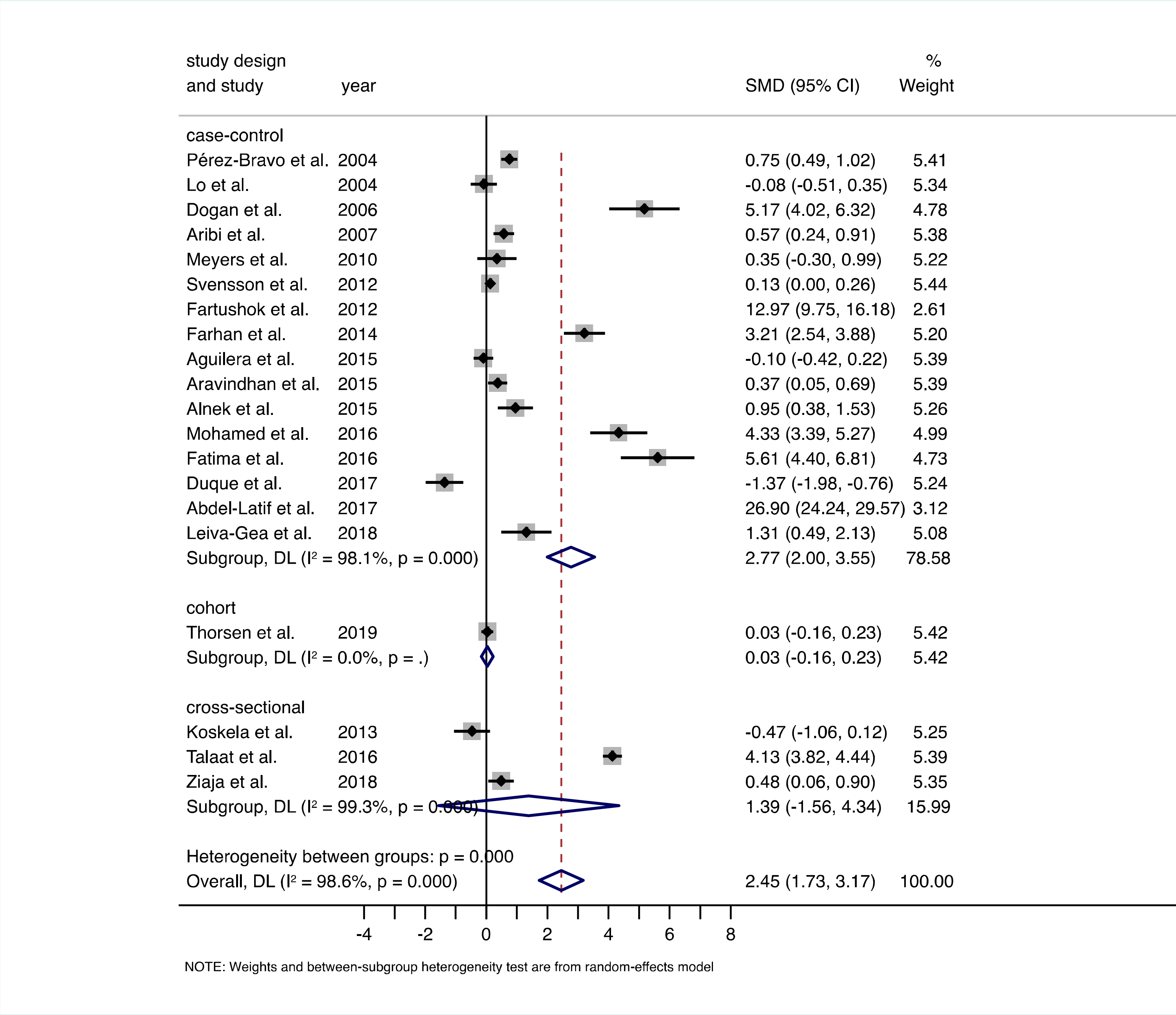

Random-effects model, inverse-variance weighting based on the DerSimonian and Laird method. Standardized mean difference (SMD) was chosen as effect size measure. An SMD>0 suggests that IL-1β levels are higher in T1DM. Diamonds indicate the overall pooled SMDs with their corresponding 95% confidence intervals (CI).

6. Meta-regression analyses

6.1. Figure S9. Effect of the covariate Sex

Bubble plot graphically representing the univariable meta-regression analysis of the potential effect of sex (% of males) on circulating IL-1β levels among patients with T1DM compared with controls.

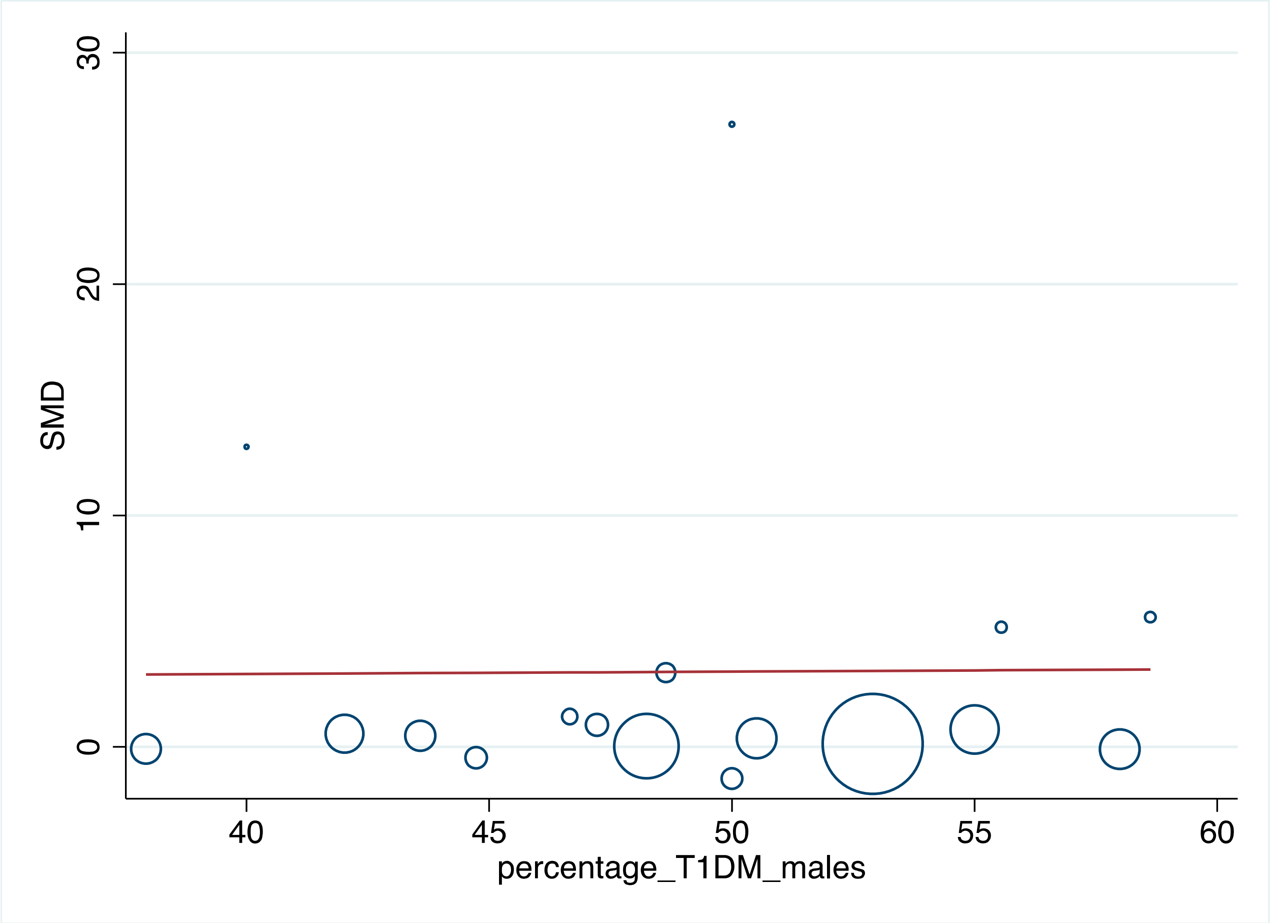

Random-effects univariable meta-regression. The red line exhibits the fitted regression line together with blue circles representing the estimates from each individual study, sized according to the precision of each estimate (the inverse of its within-study variance).

6.2. Figure S10. Effect of the covariate Risk of Bias

Bubble plot graphically representing the univariable meta-regression analysis of the potential effect of risk of bias (scored using Newcastle-Ottawa Scale) on circulating IL-1β levels among patients with T1DM compared with controls.

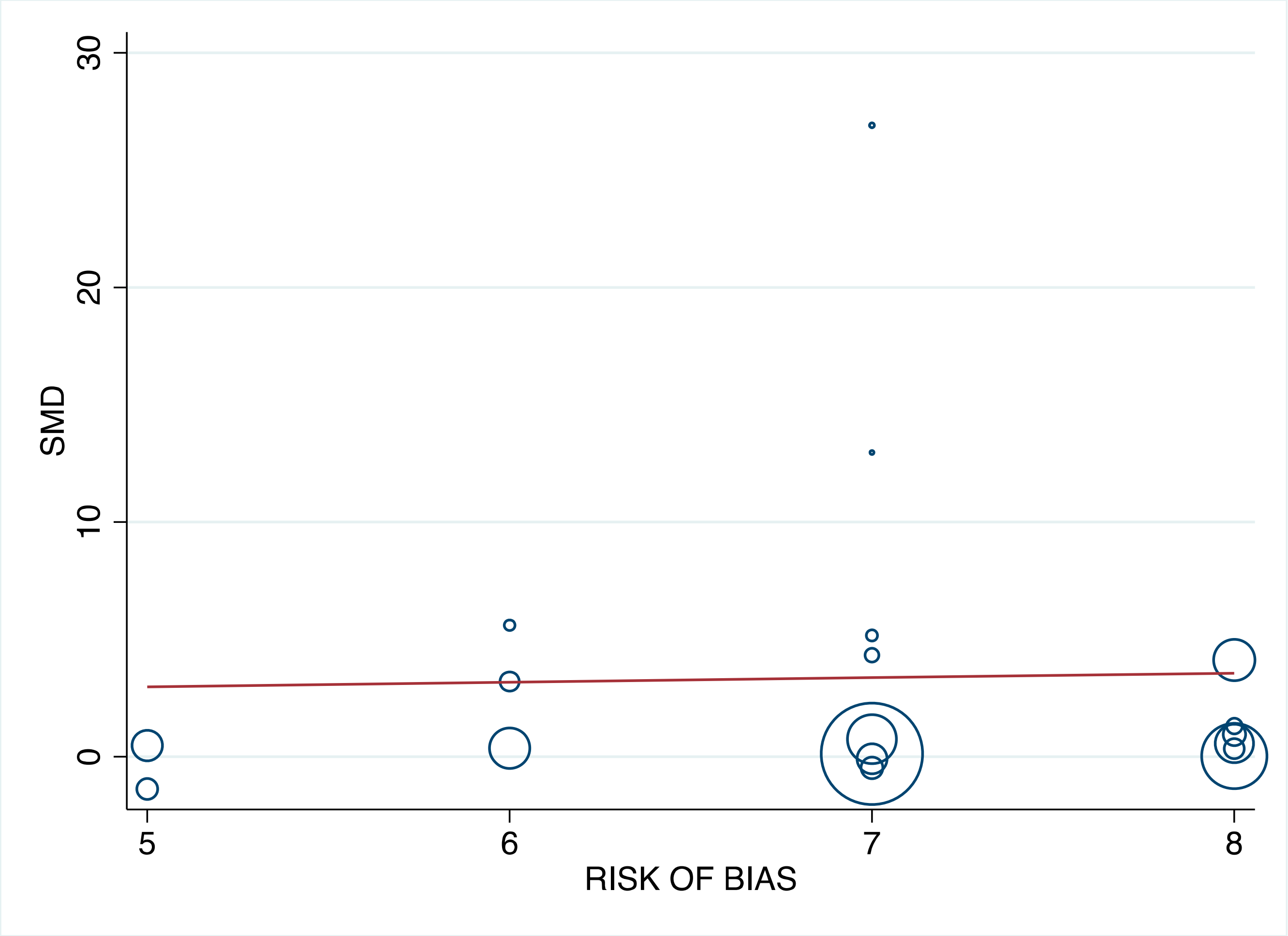

Random-effects univariable meta-regression. The red line exhibits the fitted regression line together with blue circles representing the estimates from each individual study, sized according to the precision of each estimate (the inverse of its within-study variance).

7. Table S3. Sensitivity analysis.

Sensitivity analyses evaluating the influence of individual studies on the combined results of the meta-analyses.

Sensitivity analysis ("leave-one-out" method) of the meta-analysis results, sequentially omitting one study at a time.

**Study omitted**	**Estimate**	**95% confidence intervals**	
Meta-analysis on IL-1β determination by immunoassays			
Pérez-Bravo et al. (2004)	2.608	1.825	3.392
Lo et al. (2004)	2.614	1.860	3.368
Dogan et al. (2006)	2.294	1.570	3.018
Aribi et al. (2007)	2.596	1.829	3.363
Meyers et al. (2010)	2.577	1.831	3.324
Svensson et al. (2012)	2.739	1.878	3.601
Fartushok et al. (2012)	2.153	1.438	2.868
Koskela et al. (2013)	2.621	1.875	3.366
Farhan et al. (2014)	2.396	1.666	3.126
Aguilera et al. (2015)	2.631	1.866	3.395
Aravindhan et al. (2015)	2.611	1.841	3.380
Alnek et al. (2015)	2.547	1.798	3.295
Mohamed et al. (2016)	2.333	1.608	3.059
Fatima et al. (2016)	2.272	1.550	2.994
Talaat et al. (2016)	2.128	1.521	2.736
Duque et al. (2017)	2.659	1.921	3.396
Abdel-Latif et al. (2017)	1.596	0.970	2.221
Leiva-Gea et al. (2018)	2.518	1.775	3.260
Ziaja et al. (2018)	2.586	1.829	3.343
Thorsen et al. (2019)	2.681	1.873	3.490
**Combined**	2.451	1.731	3.171
Meta-analysis on IL-1β determination by qRT-PCR			
Duarte et al. (2007)	.523	.220	.827
Ururahya et al. (2012)	-1.889	-2.641	-1.138
**Combined**	-.658	-3.022	1.706
Meta-analysis on IL-1β determination by Flow cytometry			
Holm et al. (2006)	1.613	-.685	3.912
Gabbay et al. (2012)	1.887	.1087	3.666
Allam et al. (2014)	.673	.1559	1.191
**Combined**	1.401	-.1949	2.998
